# Improved Inhibitor Screening Experiments by Comparative Analysis of Simulated Enzyme Progress Curves

**DOI:** 10.1371/journal.pone.0046764

**Published:** 2012-10-10

**Authors:** Fredrik Tholander

**Affiliations:** Department of Microbiology, Tumor and Cell Biology, Karolinska Institutet, Stockholm, Sweden; Albert-Ludwigs-University, Germany

## Abstract

A difficulty associated with high throughput screening for enzyme inhibitors is to establish reaction conditions that maximize the sensitivity and resolution of the assay. Deduction of information from end-point assays at single concentrations requires a detailed understanding of the time progress of the enzymatic reaction, an essential but often difficult process to model. A tool to simulate the time progress of enzyme catalyzed reactions and allows adjustment of reactant concentrations and parameters (initial concentrations, *K*_m_, *k*_cat_, *K*_i_ values, enzyme half-life, product•enzyme dissociation constant, and the rate constant for the reversed reaction) has been developed. This tool provides comparison of the progress of uninhibited versus inhibited reactions for common inhibitory mechanisms, and guides the tuning of reaction conditions. Possible applications include: analysis of substrate turnover, identification of the point of maximum difference in product concentration (Δ_max_[*P*]) between inhibited and uninhibited reactions, determination of an optimal observation window unbiased for inhibitor mechanisms or potency, and interpretation of observed inhibition in terms of true inhibition. An important observation that can be utilized to improve assay signal strength and resolution is that Δ_max_[*P*] occurs at a high degree of substrate consumption (commonly >75%) and that observation close to this point does not adversely affect observed inhibition or IC_50_ values.

## Introduction

High-throughput screening (HTS) is a complex task involving diverse aspects ranging from buffer optimization and enzyme characterization to robotics [1]. The choice of detection technology is another critical aspect that must be determined with awareness of the limitations associated with each method [2], *e.g.* the inner filter effect for fluorescence technologies, high substrate conversion requirement for fluorescence polarization, interference with test compounds, detection limits, and the linear range of signal responses.

In HTS for enzyme inhibitors a central concern is to design an assay with a high signal-to-background (S/B) ratio, and to determine an observation window that provides appropriate separation in read-out between hits and the control samples [3], [4]. A commonly used quantitative measure of HTS assay quality, that takes this separation and the associated standard deviations into account, is the Z-factor (Z = 1−(3σ_p_+3σ_n_)/|P-N|, were σ denotes the standard deviation of the corresponding mean of positive, P, and negative, N, controls.) [5]. To achieve an assay with a good Z-factor (*i.e.* between 0.5–1, were 1 defines an ideal assay), experimental noise should be minimized while maximizing the S/B ratio.

A common experimental condition in HTS for enzyme inhibitors is to use low substrate concentrations (*i.e.* close to *K*_m_) to avoid saturation of the active site, which would risk missing competitive inhibitors. With low substrate concentrations it often becomes necessary to allow reactions to proceed until a large proportion of substrate becomes depleted in order to obtain sufficiently high signals (*i.e.* a high S/B ratio). While such extended incubation times may obscure the effect of weak inhibitors, shorter incubation times give weaker signals that may adversely affect assay performance. Different modes of inhibition (*e.g*. uncompetitive and non-competitive) further complicates data interpretation and assay design. A further difficulty is that the underlying theory, which is based on rate-law equations for *initial* reaction velocity, becomes violated at extended reaction times and thus complicates data interpretation, particularly the relation between observed and true inhibitor potency [6].

In experimental deduction of kinetic parameters, the initial reaction rate at different substrate concentrations is measured and data obtained fitted to the Michaelis-Menten (MM) rate law equation (**Equations S1,** equation 1). In practice, initial reaction rates can only be approximated since the real measurable quantity represents a concentration at a given time-point (*i.e.* samples are taken along a reaction progress curve). With small enough time intervals, which is commonly used when determining kinetic parameters, the approximation improves, and for many experiments this is not a problem. However, conditions such as those commonly applied to HTS for enzyme inhibitors often violate this approximation and make interpretations based on the MM equation for initial reaction velocity less reliable. Furthermore, such assays are often associated with a high enough level of substrate turnover to render the phenomenon of product inhibition (and possibly also the reversed reaction) significant, thus complicating interpretation of observed inhibition further. Consequently, interpretation of data from experiments such as HTS, as well as the design of HTS assay conditions, should ideally be founded on progress curve analysis. Since the MM rate law cannot be analytically integrated to explicitly express product concentration as a function of time and in terms of *k_cat_* and *K*_m_, this has to be achieved by numeric approaches [7], [8], [9]. Due to these issues, a tool in spreadsheet format specifically designed to simplify the analysis and design of HTS assays has been developed. The tool is simple to use and only requires knowledge in standard enzyme kinetics. It provides comparative analysis of the progress of uninhibited versus inhibited reactions for common inhibitory mechanisms and takes reaction reversibility and enzyme half-life into account. Reactions are simulated in response to adjustment of kinetic parameters and key data are automatically deduced.

## Results

### An interactive tool for simulation, comparison, and analysis of enzymatic progress curves

**Figure 1:**
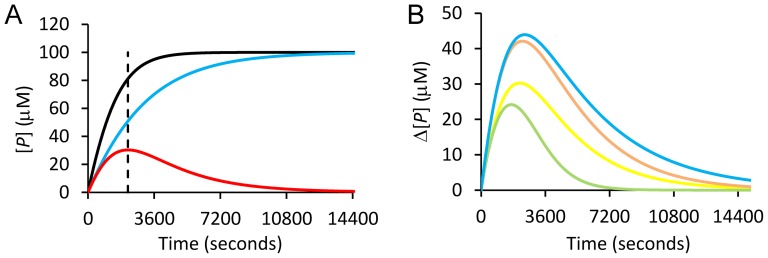
Simulated progress curves and differences between inhibited and uninhibited reactions. (**Left**) Simulated progress curves for competitive (blue trace), uninhibited (black trace) reactions, and the difference (Δ[*P*]) between the two reactions (red trace). Δ_max_[*P*] is indicated by a dashed vertical line. (**Right**) Δ[*P*] between inhibited and uninhibited enzyme reactions for competitive (yellow), uncompetitive (green), non-competitive (orange), and mixed (blue) inhibition. In the simulation tool, progress curves for all types of inhibition are shown as in the left panel. Reaction parameters and variables can be entered and the results will be directly displayed in the graphs. Entered reaction conditions were: [*S*] = *K*_m_ = 0.25*K*_mp_ = 10[*I*] = 400[*E*_o_] = 100 µM; [*P*_o_] = 0 µM; *k*_cat_ = 0.5 s^−1^; enzyme *t*_(1/2)_ = 24 hours; *K*_ic_ = *K*_iu_ = *K*_i-non_ = 5 µM for competitive, uncompetitive, and non-competitive inhibition; and *K*_iu_ = 5*K*_ic_ = 15 µM for mixed inhibition.

A tool in spreadsheet format in which progress curves of inhibited and uninhibited reversible enzyme reactions can be interactively adjusted and compared for various types of inhibition was developed. The tool can be downloaded as supplementary material (**Simulation Tool S1**), or obtained from the author. Reaction variables ([*E*_tot_], enzyme concentration; [*I*], inhibitor concentration; [*S*_o_], initial substrate concentration; [*P*_o_], initial product concentration) and parameters (*k*_cat_, turnover number; *K*_m_, Michaelis constant; *K*_p_, product·enzyme dissociation constant; *k*_−2_, rate constant for the reversed reaction; *K*_i_ values, inhibitor·enzyme dissociation constants for three modes of inhibition; *t*_(1/2)_, enzyme half life) can be adjusted and the effects of entered values are directly coupled to graphs displaying the formation of product as a function of time (**Fig. 1** **& S1**). The modes of inhibition included are; competitive (inhibitor·enzyme dissociation constant *K*_ic_), uncompetitive (inhibitor·enzyme dissociation constant *K*_iu_), and mixed inhibition (inhibitor·enzyme dissociation constants *K*_ic_ and *K*_iu_, were *K*_ic_≠*K*_iu_). This allows for non-competitive inhibition to be accounted for by setting the two inhibitor·enzyme dissociation constants for mixed inhibition to equal values (*i.e. K*_ic_ = *K*_iu_). The graphs also display the difference in product concentration between inhibited and uninhibited reactions (Δ[*P*]) as a function of time for the different types of inhibition, which is particularly useful for estimation of when the maximum difference in product concentration (Δ_max_[*P*]) occurs between uninhibited and inhibited reactions. For simple comparison, the time progress of Δ[*P*] is also shown in a separate graph for all three types of inhibition (**Fig. 1**). Additional graphs show, for each type of inhibition mechanism, Δ[*P*] as a function of inhibition (%) as the reaction proceeds, and Δ[*P*] as a function of the degree of substrate conversion (**Fig. S2**).

### Automatic deduction of key data

**Figure 2:**
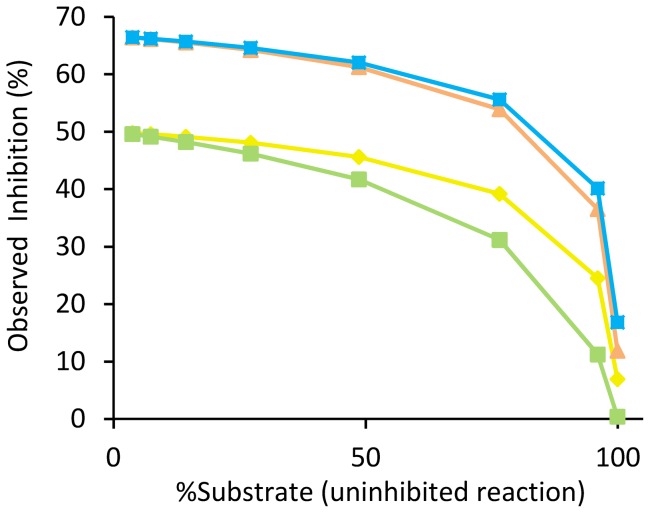
Inhibition as a function of substrate conversion. Observed inhibition (%) as a function of the degree of substrate conversion (%) of the uninhibited reference reaction for competitive (yellow), uncompetitive (green), non-competitive (orange), and mixed (blue) inhibition. In the simulation tool, time points can be entered (see **Fig. S1**) to directly study the effects in the graphs, where observed inhibition is also plotted against reaction time. Entered reaction conditions were: [*S*] = *K*_m_ = 0.25*K*_mp_ = 10[*I*] = 400[*E*_o_] = 100 µM; [*P*_o_] = 0 µM; *k*_cat_ = 0.5 s^−1^; enzyme *t*_(1/2)_ = 24 hours; *K*_ic_ = *K*_iu_ = *K*_i-non_ = 5 µM for competitive, uncompetitive, and non-competitive inhibition; and *K*_iu_ = 5*K*_ic_ = 15 µM for mixed inhibition.

**Figure 3:**
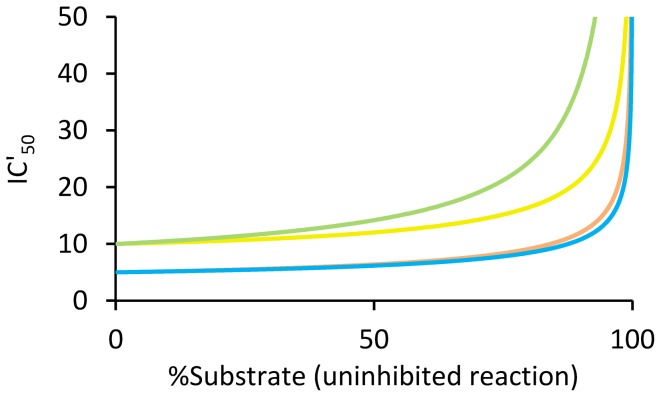
Observed inhibitor potency (IC'_50_) as a function of substrate depletion. The graph shows IC'_50_ as a function of the degree of substrate conversion of the uninhibited reference reaction for competitive (yellow), uncompetitive (green), non-competitive (orange), and mixed (blue) inhibition. In the tool, the graph is directly coupled to user adjustable reaction variables and parameters. At initial reaction conditions, IC'_50_ equals IC_50_. Entered reaction conditions were: [*S*] = *K*_m_ = 0.25*K*_mp_ = 10[*I*] = 400[*E*_o_] = 100 µM; [P_o_] = 0 µM; *k*_cat_ = 0.5 s^−1^; enzyme *t*_(1/2)_ = 24 hours; *K*_ic_ = *K*_iu_ = *K*_i-non_ = 5 µM for competitive, uncompetitive, and non-competitive inhibition; and *K*_iu_ = 5*K*_ic_ = 15 µM for mixed inhibition.

Key data are extracted from the curves and presented in table format. For each type of inhibition the time point of Δ_max_[*P*] between inhibited and uninhibited reactions, the observed inhibition (%) at that point, the associated amount of substrate conversion (%), and the value of Δ_max_[*P*] (µM) are given (**Fig. S1**). There is also a possibility to directly enter specific time-points to see the resulting Δ[*P*], the percentage substrate conversion, and the degree of inhibition (%). Entered time points are coupled to two graphs displaying the degree of inhibition (%) as a function of time and substrate conversion (**Fig. 2**). Reaction parameters are also coupled to a graph that displays observed inhibitor potency (IC'_50_) as a function of substrate consumption (**Fig. 3**). Since the tool accounts for reversible reactions, the equilibrium constant of the overall reaction (the Haldane relationship, *K*_eq_ = *V*_f_ *K*_mb_/*V*_b_ *K*_mf_) is automatically deduced.

### Simple comparative analysis of different reaction conditions

Prior to experimentation, specific reaction conditions can be studied and compared by feeding different reaction parameters into the simulation tool. With a given set of conditions ([*S*] = 2*K*_m_ and [*I*] = 2*K*_ic_) and competitive inhibition the effect of different incubation times can be studied (**Table 1**, left section). With these settings it is easy to deduced that Δ_max_[*P*] is 54 µM and occurs after 50 minutes, that this corresponds to 84% substrate conversion for the uninhibited reference reaction, and that the observed inhibition at this point is 32%. In contrast, at a time point clearly within the linear phase of the reaction (10 minutes), Δ[*P*] is 19 µM, substrate conversion 24% (uninhibited reference reaction), and the observed inhibition 40%. Since the degree of inhibition is similar at these two points, but Δ[*P*] is more than doubled at Δ_max_[*P*], a time point closer to Δ_max_[*P*] is appropriate for read-out.

**Table 1:** Observation at different time points and with different reaction conditions.

	*S*_0_ = 200 µM; *I* = 10 µM (IC_50_ = 15 µM)		*S*_0_ = 100 µM; *I* = 5 µM (IC_50_ = 10 µM)	
	Initial	Δ_max_[*P*]	Initial	Δ_max_[*P*]
Time	10 min	50 min	10 min	32 min
Δ[*P*]	19 µM	54 µM	11 µM	20 µM
Inhibition	40%	32%	32%	25%
*S* conversion	24%	84%	33%	78%

Comparison of observation at the time point of Δ_max_[*P*] and at initial reaction conditions, with two different substrate and inhibitor concentrations. Other parameters and variables: *K*_ic_ = 5 µM, *K*_m_ = 100 µM, *k*_cat_ = 0.5 s^−1^, *t*_(1/2)_ = 24 hours, *K*_p_ = 1000 µM, *k*_rev_ = 0 s^−1^, *P*_0_ = 0, *E*_tot_ = 0.25 µM. Competitive inhibition.

The effect of lowering substrate concentration to *K*_m_ and inhibitor concentration to *K*_ic_, can easily be studied while keeping other conditions fixed (**Table 1**, right section). This change results in the lowering of Δ_max_[*P*] to 20 µM after 32 minutes, with an observed inhibition of 25%, and 78% substrate consumption. After 10 minutes Δ[*P*] is 11 µM (and possibly too small to give a robust signal), the observed inhibition 32%, and substrate conversion 33%. Thus, the assay resolution becomes lower under these conditions.

### Accurate agreement with experimental data

**Figure 4:**
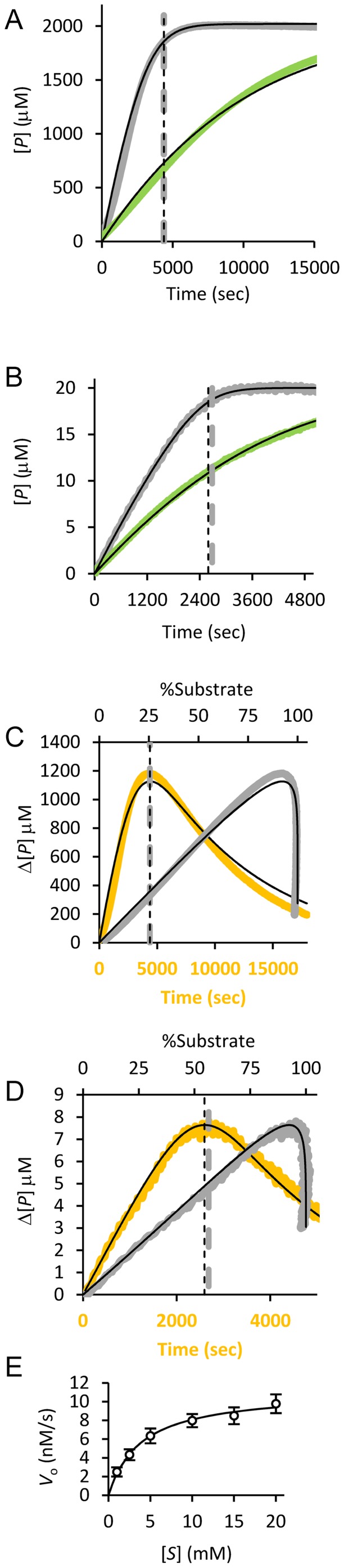
Agreement between simulated and experimental data. A–B) [*P*] as a function of time for enzyme catalyzed hydrolysis of alanine-4-nitroanilide by LTA4 hydrolase (A), and of Mca-R-P-P-G-F-S-A-F-K(Dnp)-OH by presequence peptidase (B). Reactions were performed with (green traces) and without (gray traces) the inhibitor bestatin for both enzymes. C–D) Δ[*P*] as a function of time (orange trace, lower x-axis) and as a function of substrate depletion (gray trace, upper x-axis) for LTA4 hydrolase (C) and for presequence peptidase (D). Simulated curves fit well to the experimental data (thin black lines, all panels). The observed (vertical thick dashed lines) and predicted (vertical thin dashed lines) time point of Δ_max_[*P*] are in good agreement. E) Initial reaction rate experiment with presequence peptidase and increasing [*S*]. The fitted model is shown as a black line, measured data as open circles.

Comparison with experimentally generated progress curves for LTA4H catalyzed peptide hydrolysis, with or without the competitive inhibitor bestatin, showed that simulated and experimental curves are in agreement. This suggests that predictions based on the simulation tool have significance (**Fig. 4**). By manually setting the kinetic parameters to known values (*k*_cat_ = 1 s^−1^, *K*_m_ = 1 mM and *K*_i_ = 200 nM), a good correlation between observed and simulated data was obtained; Δ_max_[*P*] was predicted to 1.23 mM and its time point to 77 minutes, which only deviates slightly from the observed Δ_max_[*P*] of 1.18 mM after 73–74 minutes. This demonstrates how a reasonable estimate of the progress curves can be directly obtained by using kinetic parameters pre-determined in an initial rate experiment, or based on literature values. It should be noted that full determination of kinetic parameters, inhibition mechanism, and error estimates, by progress curve analysis requires more data at different [S] and [I], as in initial rate experiments [10]. It should also be kept in mind that while an appropriate set of progress curve data can allow determination of *K*_m_, the underlying rate constants, *i.e.* (*k*_−1_+*k*_cat_)/*k*_1_ = *K*_m_, remains undetermined if not other types of kinetic data is available. Furthermore, as for any method relying on progress curve analysis, the linearity and range of the response must be accounted for when interpreting and comparing experimental data with simulated data.

To further evaluate the validity of the simulations, progress curves were also collected for another enzyme system, peptidolysis by presequence peptidase, with and without bestatin as an inhibitor (**Fig. 4**). Kinetic parameters were derived by least-squares model fitting. As a comparison, an experiment to determine kinetic parameters with initial reaction rates at increasing substrate concentrations was also performed (**Fig. 4**). Curve fitting gave a good data-to-model agreement, for both progress curves and the initial reaction rate experiment, and kinetic parameters derived with the two methods were very similar. Thus, *K*_m_ was determined to be 3.6 µM and *k*_cat_ to be 0.43 sec^−1^ by progress curves analysis and to be 3.9 µM and 0.45 sec^−1^ by the initial rate method. (One reason for the successful determination of *K*_m_ by the progress curve method was the possibility to use a relatively high [*S*_o_] of ∼5 *K*_m_.) Comparing the experimentally observed Δ_max_[*P*] and the time point when it occurred, with the corresponding values predicted by the tool, gave deviations of only 0.17 µM (2%) and a 90 seconds (3%), respectively.

### Simple usage

**Figure 5:**
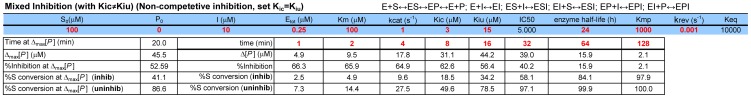
Screen dump of a subsection of the simulation tool. The tool contains three blocks (one for competitive, one for uncompetitive, and one for mixed inhibition; non-competitive inhibition is achieved by setting the two *K*_i_ values for mixed inhibition to equal values) in which the reaction parameters and variables can be set. The block for mixed inhibition is shown. Adjustable values are shown in red and are found in the second row of each block. The identities of the adjustable values are shown in the top row. The resulting IC_50_ value and overall equilibrium constant of the reversible reaction are also shown in the top row. A table presents the time point of Δ_max_[*P*] and the associated key data that result from adjustment of reaction conditions. To deduce the corresponding data at other time points, the desired values can be entered into cells of the top row of the table. In the simulation tool, changes of reaction conditions are also visualized in various graphs.

In the tool the user is presented to three differently colored blocks (for competitive, uncompetitive, and mixed inhibition) in which reactions parameters and variables can be adjusted (**Fig. 5**). Various curves are directly simulated in response to changed parameters. Adjustable cells/values are shown in red and other cells are locked for editing. Each block also has a table presenting important key data (Δ_max_[*P*] along with the associated time point, % Substrate, % Inhibition, and IC'_50_) that are automatically deduced in response to adjusted parameters. The corresponding data at defined time points can also be extracted by entering time points in the column headers of an adjacent table (also coupled to graphs). This helps the user to identify an optimal window of observation. For each type of inhibition the corresponding reaction equations are given. Non-competitive inhibition is not directly included but can be accounted for setting *K*_ic_ = *K*_iu_, realizing that this is a special case of mixed inhibition.

## Discussion

A tool for the simulation and comparative analysis of enzymatic progress curves for common types of inhibition has been developed. The tool can be downloaded as supplemental material (**Simulation Tool S1**) or obtained from the author. The tool provides accurate simulation of experimental progress curves (**Fig. 4**) - given that the enzyme system under study can be approximated by the underlying model, as in any simulation approach. Reaction parameters and concentrations can be adjusted to directly observe the effects on displayed progress curves and essential data are deduced and clearly presented (**Figs. S1 &** **5**). The tool is particularly intended to support experimental design and to facilitate interpretation of data obtained in end-point assays, *e.g*. in HTS for enzyme inhibitors. In these processes the tool can be used to: study the effect of reaction conditions on the choice of observation window (see **Table 1**), tune reaction condition in favor of a particular type of inhibition, investigate the amount of substrate turn-over that can be allowed to increase the assay signal without severely affecting observed inhibition, adapt assay conditions to an enzyme with pronounced product inhibition, or to guide the selection of hit cut-off criteria while accounting for product inhibition, reaction reversibility, and substrate turn-over. Importantly, the tool has a dedicated purpose in HTS assay design, is simple to use, and only requires basic knowledge in enzyme kinetics. This is in contrast to other more advanced simulation software for more general usage, which are also considerably more complex, *e.g.* Dynafit [11], [12], Fitsim/Kinsim [8], [9], and Gepasi [13], [14] with its successor Copasi [15].

A general observation that emerges when using the simulation tool is that Δ_max_[*P*] occurs at the end of the linear phase of the uninhibited reference reaction (*cf*. **Figs. 1** **& S1**). The associated degree of substrate consumption is normally above 70% and is rather insensitive to changes in substrate and inhibitor concentrations. Furthermore, the observed inhibition does not significantly deviate from initial conditions until the substrate is almost completely depleted (>80%). An ideal point of observation should therefore be within a window of about 50–75% substrate consumption, which is in contrast to the common recommendation of <20% substrate depletion for determination of kinetic parameters.

Since interpretation of observed inhibition in terms of true inhibition can become confused at such high levels of substrate depletion, the tool clearly visualizes observed inhibition (%) and inhibitor potency (IC'_50_) as a function of substrate consumption (**Figs. 2** **–** **3**). These graphs help to deduce when and to what extent observed inhibition starts to significantly deviate from inhibition at initial reaction conditions. Importantly, the tool also accounts for product inhibition and enzyme inactivation in these calculations. Deviations between true and observed inhibition are generally small up to a high degree of substrate depletion (**Fig. 2** **–** **3**), and become stable over an even wider range at increased inhibitor and substrate concentrations. Taken together, this information is particularly useful for data evaluation, *e.g.* to decide the degree of observed inhibition to be taken as hit criteria in an HTS assay with high substrate consumption and significant product inhibition, or to transform observed inhibition (IC'_50_) to true inhibition (IC_50_) at specific reaction conditions.

Comparing different modes of inhibition at corresponding reaction conditions indicates that non-competitive and mixed inhibition exhibit the largest Δ[*P*] values and thus the highest degree of observed inhibition throughout the whole reaction time course (**Fig. 1** **& S2**). The fact that non-competitive and mixed inhibitors have two *K*_i_ values augmenting each other explains this effect. Consequently, identification of non-competitive and mixed inhibitors are generally favored.

It is well known that a substrate concentration well above *K*_m_ increases the effect of uncompetitive relative to competitive inhibitors, while a substrate concentration below *K*_m_ has the opposite effect. By minimizing the least-square difference between progress curve for competitive and uncompetitive inhibition with identical *K*_i_ values the tool shows that the difference in the reaction progress between these two mechanisms is minimized at an initial substrate concentration of about 1.6 *K*_m_. It is also clear that a longer reaction time favors the detection of inhibitors with an element of competitive inhibition (*i.e.* competitive, mixed, and non-competitive inhibition, which is a special case of mixed inhibition with *K*_ic_ = *K*_iu_), since the observed inhibition for uncompetitive inhibition falls off first (**Fig. 2**). A low substrate concentration in combination with high substrate consumption therefore bias assay conditions toward detection of inhibitors with an element of competitive inhibition.

As outlined above, an observation window close to the time point of Δ_max_[*P*] is optimal. An additional advantage is that signal strength is increased and assays with weak signals can be made useable. A high degree of substrate depletion is also beneficial for certain fluorescence polarization-based assays, *e.g.* the IMAP assay [16]. Importantly, IC'_50_ at the time point of Δ_max_[*P*] only deviates slightly from IC_50_ at initial reaction conditions (**Fig. S3**). An observation window close to this point is also equally suited for measurement of either substrate depletion or product formation. The price is that a lower degree of inhibition should be used as cut-off compared to an assay relying on conditions closer to initial reaction velocity (**Fig. 2**), which further favors observation close to Δ_max_[*P*] because resolution is maximized simultaneously with Δ[*P*]. It is also important to realize that, once the standard deviation of the assay read-out has been optimized, increasing Δ[*P*] is the only way to improve the Z-factor, because of its asymptotic behavior (**Fig. S4**). For instance, with a coefficient of variation of 10%, the upper limit of the Z-factor is 0.7 and an S/B ratio of 3 gives a Z-factor of 0.4. However, increasing the S/B ratio above 4 gives a Z-factor above 0.5. The nature of the Z-factor thus emphasizes the importance of a large Δ[*P*].

In summary, HTS assays are often designed with a set of common rules of thumb in mind, *e.g.* no more than 20 percent substrate conversion, or substrate conversion should be within the linear range, or substrate concentration must equal *K*_m_. Such rules may sometimes limit the successful design of experiments by giving low signal windows. Many of the rules that guide the set-up of HTS assays stem from a fear of violating MM conditions and the underlying assumptions for steady state kinetics. Comparative analysis of progress curves of uninhibited versus inhibited reactions helps to better understand when, and to what extent, these concerns should be accounted for and also helps in the final data interpretation. The tool presented here aids in this process and provides accurate simulations of experimental progress curves.

## Materials and Methods

### Recombinant protein, reagents and chemicals

Purified LTA4 hydrolase was a gift from Dr. Agnes Rinaldo Mattis, Karolinska Institute. Presequence peptidase was a gift from Prof. Elzbieta Glaser, Stockholm University. Mca-R-P-P-G-F-S-A-F-K(Dnp)-OH was from R&D Systems, UK. Alanine-4-nitroanlide and other standard chemicals were from Sigma-Aldrich, Sweden.

### Simulation of progress curves

To simulate reaction progress curves expressing product concentration as a function of time, the reversible MM rate law for initial reaction velocity (**Equations S1,** equation 2) was numerically integrated according to Euler's method with an integration time step of 1 second (**Equations S1,** equation 3). A function to estimate enzyme half life was also included as a mechanism-free model that accounts for enzyme inactivation by reducing its concentration with respect to its half-life (**Equations S1,** equation 4). Because product inhibition and the reversed reaction may be prevalent as product accumulates, these phenomena were also taken into account in all rate equations (**Equations S1,** equations 2 & 5–7). For inhibited reaction progress curves, the same approach was applied on the rate laws for the three common types of inhibition (**Equations S1,** equations 5–7). Additional equations used in the simulation tool are shown in **Equations S1** (equations 8–13).

The underlying assumption is that the rapid equilibrium approximations holds true throughout the complete reaction and that the reactions are pseudo-first-order in [*S*]. Even though the inherent pseudo-first-order assumption that [*S*]≫[*E*] becomes violated as [*S*] approaches [*E*], the associated error is normally small (**Fig. S5**). Moreover, HTS assays are typically performed with [*E*] at orders of magnitude lower than [*S*]. A level with [*S*] approaching [*E*] is thus equivalent with almost complete substrate depletion and will therefore contribute very weakly to the overall reaction rate. For the reversed reaction, the equivalent condition is most pronounced at the very beginning of the reaction. However, since the product level at this stage is very low the overall contribution to the reaction rate will be small. Another reason to work with very low [*E*] is imposed by the fact that IC_50_ values lower than one-half of the [*E*] cannot be measured. Since compounds are typically screened at 1–100 µM it is common to work with [*E*] around 100 nM, which in most cases is much lower than [*S*].

Furthermore, since many enzyme systems deviate from MM kinetics (*e.g.* due to substrate inhibition or activation, random pathways, or allosteric effects) [17] and because accuracy is limited by experimental noise, the limitations of underlying models can be neglected. More sophisticated methods for numerical integration are also not justified for the same reasons. For instance, integration using the Runge-Kutta method only generated extremely small differences, compared to the method applied, that definitely are within the limits of experimental noise.

### Experimental progress curves

To compare the simulated curves with experimentally obtained data, progress curves for LTA4H-catalyzed hydrolysis of alanine-4-nitroanilide, with and without inhibitor, were collected. LTA4H catalyses the hydrolysis of alanine-4-nitroanilide into alanine and nitroaniline, a reaction easily monitored by light absorbance spectroscopy. Uninhibited reaction mixtures contained 50 mM Tris pH7.5, 100 mM NaCl, 1 µM LTA4H, and 2 mM alanine-4-nitroanilide in a total volume of 75 µl. For inhibited reactions, 1 µM of the competitive inhibitor bestatin [18] was also included. Reactions were started by addition of alanine-4-nitroanilide and the formation of 4-nitroaniline was monitored spectrophotometrically at 430 nm for 4 hours. Control reactions without enzyme were also performed.

Progress curves were also collected for presequence peptidase (a peptidase of the pitrilysin family [19]) with Mca-Arg-Pro-Pro-Gly-Phe-Ser-Ala-Phe-Lys(Dnp)-OH (where Mca denotes (7-methoxycoumarine-4-yl)acetyl; Dnp denotes 2,4-Dinitrophenyl) as substrate. The fluorescence of the Mca moiety of the substrate is efficiently quenched by resonance energy transfer to the nearby Dnp moiety, and product formation is monitored as the increase in fluorescence intensity with excitation at 320 nm and emission at 405 nm. Since bestatin is known to be a transition state analogue for peptide bond hydrolysis, it was tested and found to be a weak inhibitor of presequence peptidase. Progress curves with and without bestatin (500 mM) was therefore collected. Other reaction constituents were: 50 mM HEPES pH 8.2, 20 µM substrate, 25 nM enzyme and 10 mM MgCl_2_. Substrate was added last to start the reactions.

For presequence peptidase, a set of initial velocity reaction experiments with substrate concentration ranging from 1–20 µM was also performed. Other conditions were as for the progress curve experiment.

### Fitting of models to experimental data

To perform data fitting, the sum of the squared errors between model and data were minimized using the Solver add-in bundled with excel [20]. This allows automatic minimization of a target cell containing the sum of the squared errors by changing the values of cells containing model parameters.

## Supporting Information

Equations S1 **Equations used in the simulation tool.**
(PDF)

Figure S1 **Screen dump from the simulation tool.** The user can modify parameters and reaction variables (adjustable values in red bold type) for the different types of inhibition (competitive, uncompetitive, and mixed inhibition; non-competitive inhibition is achieved by setting *K*_ic_ = *K*_iu_) and immediately observe the effects in various graphs. The following graphs are generated: reaction progress curves for inhibited and uninhibited reactions and the associated difference for each type of inhibition (upper row of three graphs), differences between inhibited and uninhibited reactions for the given modes of inhibition (first graph in lower row), difference as a function of substrate conversion for the given modes of inhibition (second graph in lower row), difference as a function of observed inhibition for the given modes of inhibition (third graph in lower row), observed IC_50_ (IC'_50_) as a function of substrate conversion for the given modes of inhibition (fourth graph in lower row), observed inhibition as a function of substrate conversion and time for the given modes of inhibition (upper right, two graphs). For the latter two graphs, the points plotted are governed by user-entered time-points. Essential key data are deduced from the simulated progress curves and presented in table format for each type of inhibition.
(TIF)

Figure S2 **Δ[** ***P*** **] between inhibited and uninhibited reactions as a function of substrate conversion (left) or observed inhibition (right) for four types of inhibition: competitive (yellow trace), uncompetitive (green trace), non-competitive (orange trace), and mixed (blue trace).** In the simulation tool, the graphs are directly coupled to user entered reaction parameters and variables. Entered reaction conditions were: [*S*] = *K*_m_ = 0.25*K*_mp_ = 10[*I*] = 400[*E*_o_] = 100 µM; [*P*_o_] = 0 µM; *k*_cat_ = 0.5 s^−1^; enzyme *t*_(1/2)_ = 24 hours; *K*_ic_ = *K*_iu_ = *K*_i-non_ = 5 µM for competitive, uncompetitive, and non-competitive inhibition; and *K*_iu_ = 5*K*_ic_ = 15 µM for mixed inhibition. Substrate conversion refers to the uninhibited reference reaction (**left**). Note the reversed x-axis (**right**).
(TIF)

Figure S3 **Δ[** ***P*** **] between inhibited and uninhibited reactions for competitive (yellow), uncompetitive (green), non-competitive (orange), and mixed (blue) inhibition as a function of IC'_50_ (observed IC_50_ value).** At initial reaction conditions, IC'_50_ equals IC_50_. In the tool, the graph is directly coupled to user adjustable reaction variables and parameters. Entered reaction conditions were: [*S*] = *K*_m_ = 0.25*K*_mp_ = 10[*I*] = 400[*E*_o_] = 100 µM; [*P*_o_] = 0 µM; *k*_cat_ = 0.5 s^−1^; enzyme *t*_(1/2)_ = 24 hours; *K*_ic_ = *K*_iu_ = *K*_i-non_ = 5 µM for competitive, uncompetitive, and non-competitive inhibition; and *K*_iu_ = 5*K*_ic_ = 15 µM for mixed inhibition.
(TIF)

Figure S4 **Asymptotic behaviour of the Z-factor.** The Z-factor is plotted as a function of the S/B ratio at different coefficients of variation (CV). The asymptotes are shown as horizontal dashed lines. Corresponding curves, asymptotes, and CV values are in identical colors. For a specific CV the Z-factor can only be improved by increasing the S/B ratio, with an upper bond defined by the asymptote.
(TIF)

Figure S5 **Differences between progress curves generated from pseudo-first order and second order rate equations.** Curves generated from the pseudo-first-order model are shown as thick lines and curves generated with the second-order model are shown as thin lines. **A**) Progress curves (solid lines) are plotted against the left Y-axis and the differences between the models (dotted lines) are plotted against the right Y-axis. Progress curves were generated by numeric integration of equation a and b for different *K*_m_ values, as indicated in the graph. Other variables and parameters were as follows: [*S*_o_] = 100 µM, [*E*_tot_] = 100 nM and *k*_cat_ = 0.5 s^−1^. Differences between the two models are below 1% for the *K*_m_ values tested. **B**) Progress curves (solid lines, left Y-axis) were generated by numeric integration of equation a and b for different values of [*S*_o_], as indicated in the graph, and with other parameters as follows: *K*_m_ = 20 µM, [*E*_tot_] = 100 nM and *k*_cat_ = 0.5 s^−1^. Differences between the two models (dotted lines, right Y-axis) are below 3% for the values tested. The results shown in A) and B) demonstrate that differences between progress curves generated from pseudo-first order and second order rate equations are small at conditions normally applied in HTS. The differences are of the same magnitude or smaller as experimental noise and should therefore only have a limited effect on the quality of predictions made by the tool. 1. Morrison JF (1969) Kinetics of the reversible inhibition of enzyme-catalysed reactions by tight-binding inhibitors. Biochim Biophys Acta 185: 269–286.
(TIF)

Simulation Tool S1 **The simulation tool in Microsoft Excel binary format.**
(XLSB)
