# Supplementary material for: Improved Inhibitor Screening Experiments by Comparative Analysis of Simulated Enzyme Progress Curves
Source: PLoS One. 2012 Oct 10;7(10):e46764. doi: 10.1371/journal.pone.0046764 (PMC3468632; doi:10.1371/journal.pone.0046764)
Supplement: Equations S1 — Equations used in the simulation tool. (PDF) [file pone.0046764.s001.pdf]

Supplement

Equations S1

$$V = \frac{d[P]}{dt} = -\frac{d[S]}{dt} = \frac{k_{\text{cat}}[S][E_{\text{tot}}]}{[S] + K_{\text{m}}}$$

Equation 1. The Michaelis-Menten rate-law for initial reaction velocity. k_{cat}, turnover number; [S], substrate concentration, [E_{tot}], total enzyme concentration; K_{m}, Michaelis constant.

$$V = \frac{k_{\text{cat}}[S][E_{\text{tot}}]}{[S] + K_{\text{m}}(1 + \frac{[P]}{K_{mP}})} - \frac{k_{-2}[P][E_{\text{tot}}]}{[P] + K_{\text{mp}}(1 + \frac{[S]}{K_m})}$$

Equation 2. The reversible Michaelis-Menten rate-law for initial reaction velocity. k_{cat}, turnover number for the forward reaction; [S], substrate concentration, [E_{tot}], total enzyme concentration; [P], product concentration; K_{m}, substrate concentration giving V=0.5V_{max} in forward direction at [P]=0; K_{mp}, product concentration giving V=0.5V_{max} in reverse direction at [S]=0; k_{-2}, turnover number for the reverse reaction

$$[P]_{i+\Delta t} = [P]_i + \frac{d[P]_i}{dt}\Delta t$$

Equation 3. Equation for numeric integration of the initial reaction velocity (V_{o}). $[P]_{i+\Delta t}$, product concentration at time point $i+\Delta t$; $[P]_i$, product concentration at time point i; $\frac{d[P]_i}{dt}$, reaction velocity at time point i; Δt, the integration time step.

$$[E] = [E_{\text{o}}]\left(\frac{1}{2}\right)^{t/t_{(1/2)}}$$

Equation 4. Equation expressing the decay of enzyme as a function of time with respect to its half-life. [E_{o}], enzyme concentration at time zero; t, time; $t_{(1/2)}$, enzyme half-life.

$$V = \frac{k_{\text{cat}}[S][E_{\text{tot}}]}{[S] + K_{\text{m}}(1 + \frac{[P]}{K_{mP}} + \frac{[I]}{K_{ic}})} - \frac{k_{-2}[P][E_{\text{tot}}]}{[P] + K_{\text{mp}}(1 + \frac{[S]}{K_m} + \frac{[I]}{K_{ic}})}$$

Equation 5. Reversible Michaelis-Menten equation with competitive inhibition. [I], inhibitor concentration; K_{ic}, enzyme·inhibitor dissociation constant for competitive inhibition; other notations as in equation 2.

$$V = \frac{k_{\mathrm{cat}}[S][E_{\mathrm{tot}}]}{[S](1+\frac{[I]}{K_{iu}}) + K_{\mathrm{m}}(1+\frac{[P]}{K_{mP}})} - \frac{k_{-2}[P][E_{\mathrm{tot}}]}{[P](1+\frac{[I]}{K_{iu}}) + K_{\mathrm{mp}}(1+\frac{[S]}{K_m})}$$

Equation 6. Reversible Michaelis-Menten equation with uncompetitive inhibition. K_{iu}, enzyme·inhibitor dissociation constant for uncompetitive inhibition; other notations as in equation 2.

$$V = \frac{k_{\mathrm{cat}}[S][E_{\mathrm{tot}}]}{[S](1+\frac{[I]}{K_{iu}}) + K_{\mathrm{m}}(1+\frac{[P]}{K_{mP}}+\frac{[I]}{K_{ic}})} - \frac{k_{-2}[P][E_{\mathrm{tot}}]}{[P](1+\frac{[I]}{K_{iu}}) + K_{\mathrm{mp}}(1+\frac{[S]}{K_m}+\frac{[I]}{K_{ic}})}$$

Equation 7. Reversible Michaelis-Menten equation with mixed inhibition. Notations as in equations 2 & 5-6. Note that non-competitive inhibition is obtained by setting $K_{\mathrm{iu}}=K_{\mathrm{ic}}$.

$$IC'_{50} = [I] \times \frac{[P_{inh}]}{[P_{uninh}] - [P_{inh}]}$$

Equation 8. Inhibitor concentration giving 50% observed inhibition. $[P_{inh}]$, product concentration of the inhibited reaction; $[P_{uninh}]$, product concentration of the uninhibited reference reaction; other notations as in equation 5. At initial reaction conditions, IC'_{50} equals IC_{50}.

$$\%Inhibition = 100 \times \left(\frac{[P_{uninh}] - [P_{inh}]}{[P_{uninh}]}\right)$$

Equation 9. Observed inhibition (%). Notations as in equation 8.

$$\%Substrate = 100 \times \left(\frac{[P] - [P_o]}{[S_o]}\right)$$

Equation 10. Degree of substrate depletion (%). $[P]$, accumulated product concentration; $[P_{\mathrm{o}}]$, product concentration at time zero; $[S_{\mathrm{o}}]$, substrate concentration at time zero.

$$IC_{50} = K_{\mathrm{ic}}\left(1+\frac{[S]}{K_{\mathrm{m}}}+\frac{[P]}{K_{\mathrm{mp}}}\right)$$

Equation 11*. Relation between IC_{50} and K_{ic} for competitive inhibition and product inhibition. Notations as in equations 2 & 5.

$$IC_{50} = K_{\mathrm{iu}}\left(1+\frac{K_{\mathrm{m}}}{[S]}+\frac{K_{\mathrm{m}}[P]}{K_{\mathrm{mp}}[S]}\right)$$

Equation 12*. Relation between IC_{50} and K_{iu} for uncompetitive inhibition and product inhibition. Notations as in equations 2 & 6.

$$IC_{50} = \left(S + K_m + \frac{K_\mathrm{m}[P]}{K_\mathrm{mp}}\right) \div \left(\frac{[S]}{K_\mathrm{iu}} + \frac{K_\mathrm{m}}{K_\mathrm{ic}}\right)$$

Equation 13*. Relation between IC_{50} and K_i values for mixed inhibition and product inhibition. Notations as in equations 2 & 5-6.

*Equations 11-13 were deduced by setting $0.5V = V(\mathrm{IC}_{50})$ with the expression for V from equation 2 to the left and the expression for V (with $[I]$ set to IC_{50}) from each of the equations 5-7 to the right. This gave 3 equations which were solved for IC_{50}, similar to Cheng and Prusoff [1].

References

1. Cheng, Y. & Prusoff, W.H. Relationship between the inhibition constant (Ki) and the concentration of inhibitor which causes 50 per cent inhibition (I50) of an enzymatic reaction. *Biochem Pharmacol* **22**, 3099-3108 (1973).
