# Supplementary figures and images for: Improved Inhibitor Screening Experiments by Comparative Analysis of Simulated Enzyme Progress Curves
Source: PLoS One. 2012 Oct 10;7(10):e46764. doi: 10.1371/journal.pone.0046764 (PMC3468632; doi:10.1371/journal.pone.0046764)

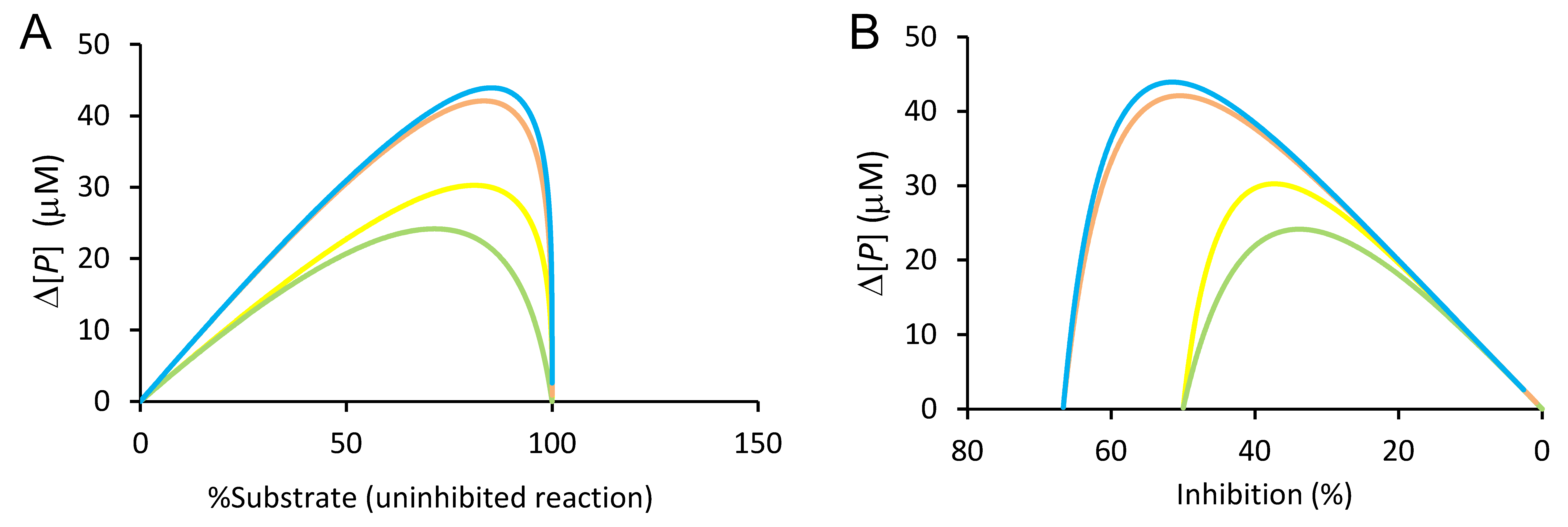

Supplement: Figure S2 — Δ[ P ] between inhibited and uninhibited reactions as a function of substrate conversion (left) or observed inhibition (right) for four types of inhibition: competitive (yellow trace), uncompetitive (green trace), non-competitive (orange trace), and mixed (blue trace). In the simulation tool, the graphs are directly coupled to user entered reaction parameters and variables. Entered reaction conditions were: [S] = K m = 0.25K mp = 10[I] = 400[E o] = 100 µM; [P o] = 0 µM; k cat = 0.5 s−1; enzyme t (1/2) = 24 hours; K ic = K iu = K i-non = 5 µM for competitive, uncompetitive, and non-competitive inhibition; and K iu = 5K ic = 15 µM for mixed inhibition. Substrate conversion refers to the uninhibited reference reaction (left). Note the reversed x-axis (right). (TIF) [file pone.0046764.s003.tif]

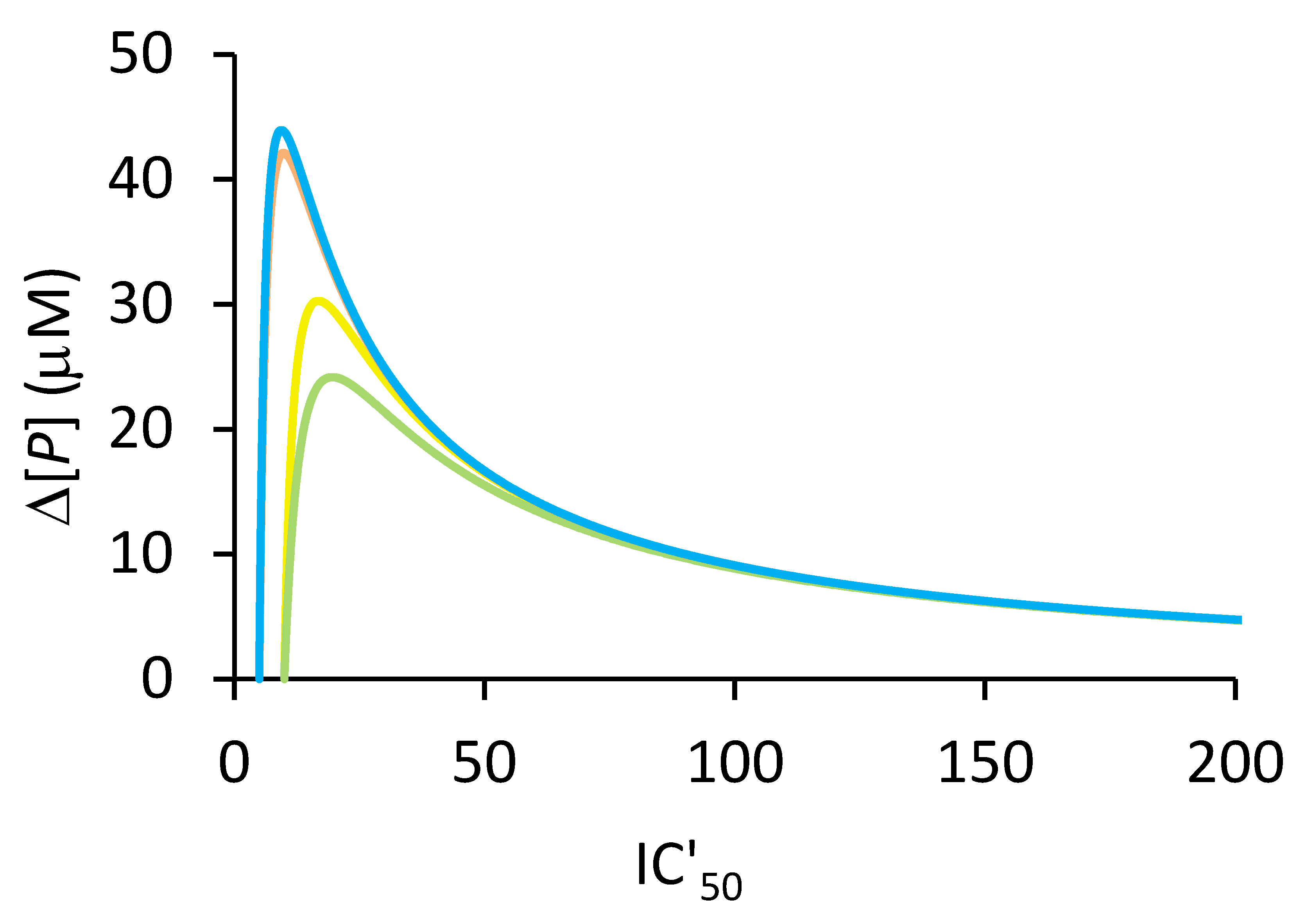

Supplement: Figure S3 — Δ[ P ] between inhibited and uninhibited reactions for competitive (yellow), uncompetitive (green), non-competitive (orange), and mixed (blue) inhibition as a function of IC'50 (observed IC50 value). At initial reaction conditions, IC'50 equals IC50. In the tool, the graph is directly coupled to user adjustable reaction variables and parameters. Entered reaction conditions were: [S] = K m = 0.25K mp = 10[I] = 400[E o] = 100 µM; [P o] = 0 µM; k cat = 0.5 s−1; enzyme t (1/2) = 24 hours; K ic = K iu = K i-non = 5 µM for competitive, uncompetitive, and non-competitive inhibition; and K iu = 5K ic = 15 µM for mixed inhibition. (TIF) [file pone.0046764.s004.tif]

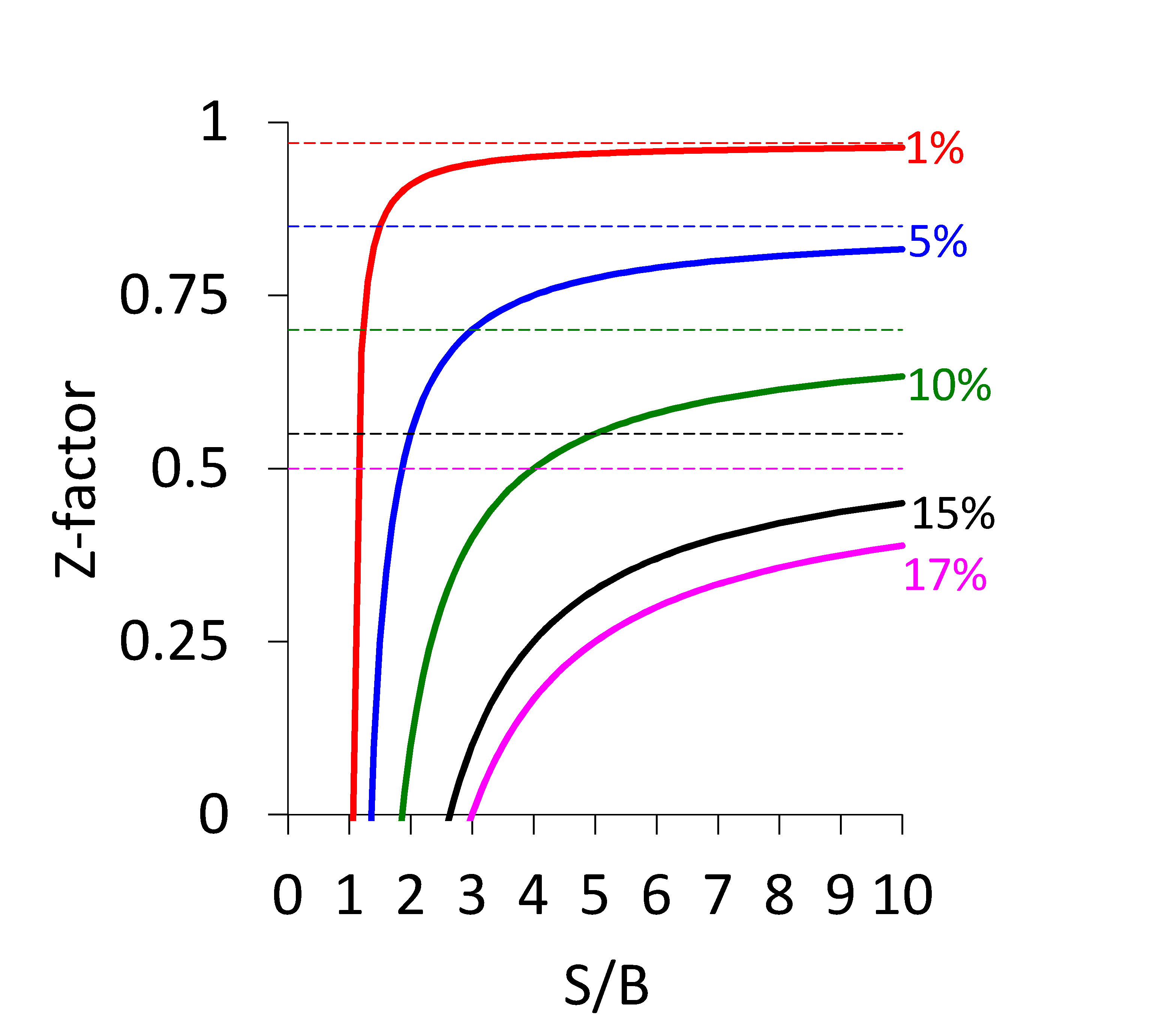

Supplement: Figure S4 — Asymptotic behaviour of the Z-factor. The Z-factor is plotted as a function of the S/B ratio at different coefficients of variation (CV). The asymptotes are shown as horizontal dashed lines. Corresponding curves, asymptotes, and CV values are in identical colors. For a specific CV the Z-factor can only be improved by increasing the S/B ratio, with an upper bond defined by the asymptote. (TIF) [file pone.0046764.s005.tif]

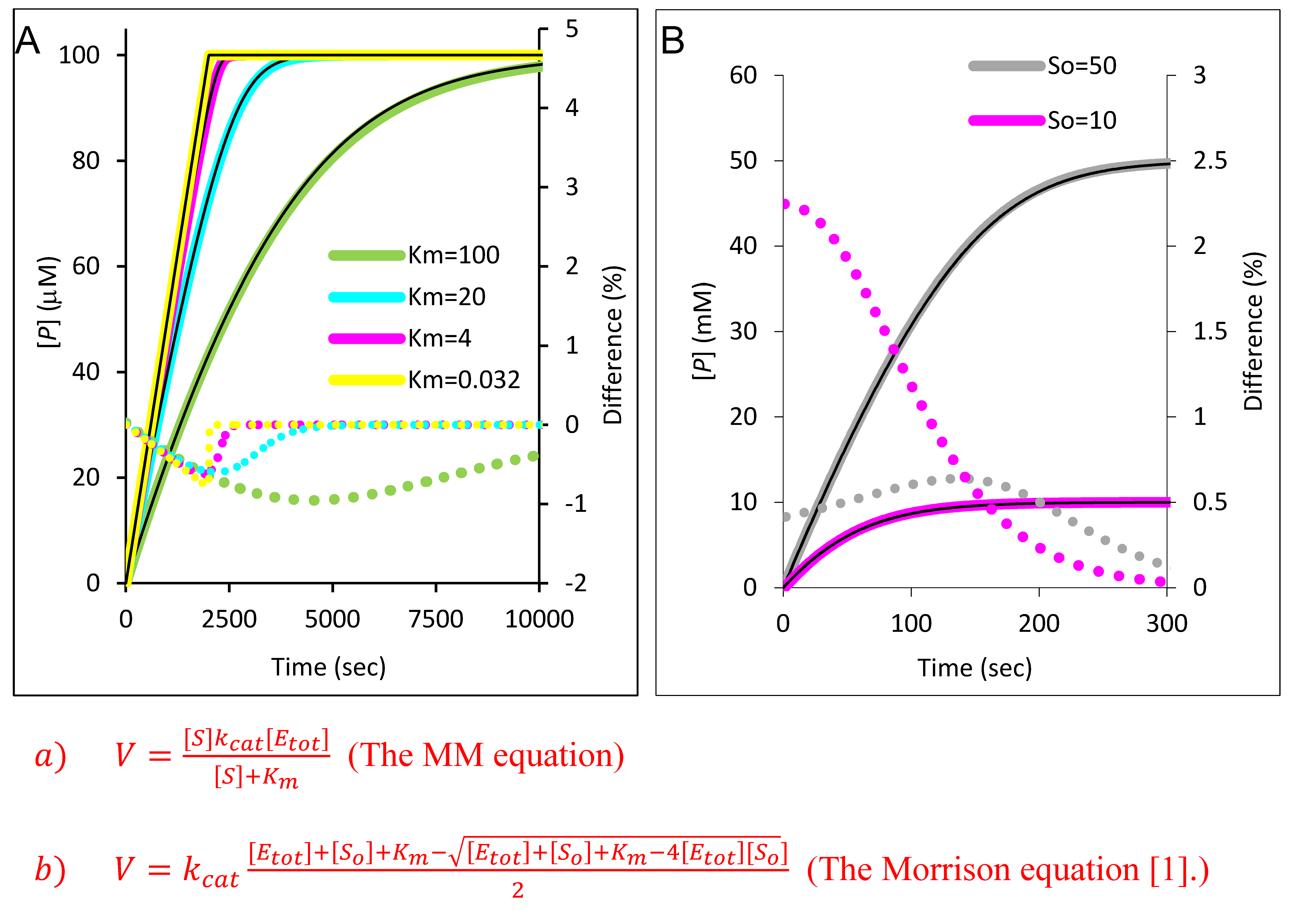

Supplement: Figure S5 — Differences between progress curves generated from pseudo-first order and second order rate equations. Curves generated from the pseudo-first-order model are shown as thick lines and curves generated with the second-order model are shown as thin lines. A) Progress curves (solid lines) are plotted against the left Y-axis and the differences between the models (dotted lines) are plotted against the right Y-axis. Progress curves were generated by numeric integration of equation a and b for different K m values, as indicated in the graph. Other variables and parameters were as follows: [S o] = 100 µM, [E tot] = 100 nM and k cat = 0.5 s−1. Differences between the two models are below 1% for the K m values tested. B) Progress curves (solid lines, left Y-axis) were generated by numeric integration of equation a and b for different values of [S o], as indicated in the graph, and with other parameters as follows: K m = 20 µM, [E tot] = 100 nM and k cat = 0.5 s−1. Differences between the two models (dotted lines, right Y-axis) are below 3% for the values tested. The results shown in A) and B) demonstrate that differences between progress curves generated from pseudo-first order and second order rate equations are small at conditions normally applied in HTS. The differences are of the same magnitude or smaller as experimental noise and should therefore only have a limited effect on the quality of predictions made by the tool. 1. Morrison JF (1969) Kinetics of the reversible inhibition of enzyme-catalysed reactions by tight-binding inhibitors. Biochim Biophys Acta 185: 269–286. (TIF) [file pone.0046764.s006.tif]
